# Predictors of Cardiovascular Autonomic Neuropathy Onset and Progression in a Cohort of Type 1 Diabetic Patients

**DOI:** 10.1155/2018/5601351

**Published:** 2018-03-05

**Authors:** M. Matta, A. Pavy-Le Traon, S. Perez-Lloret, C. Laporte, I. Berdugo, N. Nasr, H. Hanaire, J. M. Senard

**Affiliations:** ^1^Service de Diabétologie et maladies métaboliques, CHU de Toulouse, 1 avenue Jean Poulhès, 31059 Toulouse Cedex 9, France; ^2^Service de Neurologie, Hôpital Pierre-Paul Riquet, Place du Docteur Baylac-TSA 40031, 31059 Toulouse Cedex 9, France; ^3^Institut des Maladies Métaboliques et Cardiovasculaires, INSERM, Université de Toulouse, 1 avenue Jean Poulhès, BP 84225, 31432 Toulouse Cedex 4, France; ^4^Institute of Cardiology Research, University of Buenos Aires, National Research Council (CONICET-ININCA), Buenos Aires, Argentina; ^5^Service de Pharmacologie Clinique, Faculté de Médecine, CHU de Toulouse, 37 allées Jules Guesde, 31000 Toulouse, France

## Abstract

**Aim:**

The prevalence of cardiovascular autonomic neuropathy (CAN) in diabetes mellitus is well documented. However, the rate and predictors of both the development and progression of CAN have been less studied. Hereby, we assessed the rate and the major risk factors for CAN initiation and progression in a cohort of type 1 diabetic patients followed over a three-year period.

**Methods:**

175 type 1 diabetic patients (mean age: 50 ± 11 years; female/male: 76/99) with positive bedside screening for CAN were included and underwent 2 standardized autonomic testings using 4 standardized tests (deep breathing, Valsalva maneuver, 30/15 ratio, and changes in blood pressure during standing), separated by 3 ± 1 years. CAN staging was achieved according to the Toronto Consensus Panel on Diabetic Autonomic Neuropathy into 4 categories: absent, possible, confirmed, or severe CAN.

**Results:**

Out of the 175 patients included, 31.4% were free of CAN, 34.2% had possible CAN, 24.6% had confirmed CAN, and 9.7% exhibited severe CAN at the first assessment. Among the 103 patients with nonsevere CAN at inclusion, forty-one (39.8%) had an increase of at least one category when reassessed and 62 (60.2%) remained stable. A bivariate analysis indicated that only BMI and exposure to selective serotonin reuptake inhibitors (SSRIs) were significantly different in both groups. A multivariate analysis indicated that lower BMI (OR: 0.15, CI 95%: 0.05–0.48, *p* = 0.003) and SSRI exposure (OR: 4.18, CI 95%: 1.03–16.97, *p* = 0.04) were the sole predictors of CAN deterioration. In the 55 patients negative for CAN at the first laboratory assessment, 12 became positive at the second assessment.

**Conclusion:**

No clear predictive factor for CAN onset was identified. However, once present, CAN progression was related to low BMI and SSRI exposure.

## 1. Introduction

Cardiovascular autonomic neuropathy (CAN) is a common but often neglected diabetes mellitus complication. Based on the CAN Subcommittee of the Toronto Consensus Panel on Diabetic Neuropathy [1], CAN is defined as the impairment of cardiovascular autonomic control in patients with established diabetes mellitus following the exclusion of other causes. It is generally believed that CAN incidence is related to age, duration of diabetes, poor glycemic control, and microvascular disease [2–5]. Although unpredictable, CAN progression results in a significant cardiovascular morbidity and mortality [6].

A better understanding of predictive risk factors for CAN incidence and progression is crucial for the development of new strategies for follow-up as well as of novel therapeutic targets.

The purpose of the present study was to assess major risk factors of CAN onset and progression and to describe the time line changes in cardiac autonomic dysfunction in a cohort of type 1 diabetic patients over a mean period of 3 years.

## 2. Patients and Methods

### 2.1. Selection of Patients

175 consecutive type 1 diabetic patients attending the Department of Diabetes and Metabolism of Toulouse University Hospital between July 2003 and June 2010 and who had a positive bedside screening for CAN were included. Patients on beta-blocker treatment at selection were not included. The screening for CAN included 3 cardiovascular reflex tests described by Ewing and Clarke [7]: the heart rate (HR) response to deep breathing, which assesses beat-to-beat HR variation (R-R variation) during paced deep breathing [expiration-to-inspiration ratio (E:I)]; the initial HR response to standing expressed as the 30 : 15 ratio (ratio of the longest R-R interval (around the 30th beat) to the shortest R-R interval (around the 15th beat)) elicited by a change from the horizontal to vertical position; and the blood pressure (BP) response to standing. A single abnormal result suffices to suspect the diagnosis of possible CAN. The study was performed according to the French law for clinical research.

### 2.2. Laboratory Autonomic Testing

All patients with a positive screening for CAN underwent more elaborated tests as previously described [8]. The autonomic laboratory evaluation was based on continuous digital BP (photoplethysmography, Nexfin) and computerized ECG recordings during the following tests: paced deep breathing test (6 cycles/min), Valsalva maneuver (40 mmHg, 15 sec) performed in supine position, a 5 min stand test, and a 3 min isometric exercise (hand grip, 30% of maximal force) performed in seated position. The Ewing score (ES) was based on the following parameters: HR changes with deep breathing, 30/15 ratio, Valsalva ratio (HR max during maneuver : HR min during recovery), BP response to active standing, and diastolic BP increase to hand grip. The response to each test was considered normal (0), borderline (0.5), or abnormal (1) according to the laboratory normal values for age. Orthostatic hypotension (OH) was defined as a reduction in systolic BP of at least 20 mmHg and/or diastolic BP of at least 10 mmHg within 3 minutes of standing. The Ewing score (ES) ranging from 0 (normal) to 5 (severe autonomic failure) represents the sum of the responses to the different tests. Further assessment was performed using the Toronto Consensus classification based on previous tests except isometric hand grip: absence of CAN (no abnormal response, score 0), possible (one abnormal cardiovagal test, score 1), confirmed (at least 2 abnormal cardiovagal tests, score 2), and severe (1 abnormal cardiovagal test plus orthostatic hypotension, score 3).

### 2.3. Selection of CAN Potential Predictors

At the time of CAN screening, the following clinical characteristics of the patients were recorded: age, weight, body mass index (BMI), HbA1c level, diabetes duration, insulin treatment (subcutaneous insulin infusion or multiple daily injection), and diabetes-related complications. Diabetic nephropathy was assessed using the estimated glomerular filtration rate (eGFR), which was calculated using the MDRD equation and the urinary albumin/creatinine ratio (ACR) of a single early morning urine. Stage 1 nephropathy was considered for ACR between 30 and 299 mg/g, and stage 2 nephropathy was considered for ACR ≥ 300 mg/g and/or an eGFR < 60 ml·min^−1^ per 1.73 m^2^. Retinopathy stages included mild nonproliferative (NPR) (stage 1), moderate NPR (stage 2), and severe NPR or proliferative retinopathy and scatter laser treatment (stage 3). Knee and ankle reflexes, distal vibration perception, and pinprick tests were performed. Clinical distal peripheral neuropathy was diagnosed on at least one abnormal test. Associated cardiovascular risk factors such as arterial hypertension, hypercholesterolemia, and smoking were recorded. Silent myocardial ischemia was assessed using an exercise stress test or stress thallium myocardial perfusion imaging.

### 2.4. Statistical Analysis

Analyses were performed using STATA Version 10. Numerical variables with normal distribution are expressed as means ± SD and reported as median (interquartile range). Bivariate comparisons were performed by a chi-square test for categorical variables or by *t*-test for numerical variables (with adjustments for unequal variances when needed). A multivariate analysis was performed by logistic regression. All variables with bivariate *p* value < 0.10 were included. Numerical variables were categorized in tertiles for the exploration of the nonlinear relationship with the dependent variable. If present, the categorical variable was included in the multivariate model instead of the original numerical one. Multicolinearity was explored by correlation coefficients. Only participants with two complete and analyzable autonomic testings during the period of the study were included in the statistical analysis.

## 3. Results

### 3.1. Characteristics of the Studied Population

One hundred seventy-five (175) consecutive type 1 diabetic patients, who had a positive bedside screening for CAN (mean age: 50 ± 11 years, M/F: 1.30), were included in the study. One hundred fifty-eight out of 175 patients underwent a second complete and analyzable autonomic testing after a mean time interval of 3.0 ± 1.1 years.

The clinical and biochemical characteristics of the study population are depicted in Table 1. Mean BMI, duration of diabetes, and HbA1c level were 24.4 ± 3.0 kg/m^2^, 26 ± 11 years, and 7.9 ± 1.2%, respectively. Forty-two percent (42%) of patients were on subcutaneous insulin infusion, 12% were on intraperitoneal insulin infusion, and 46% received multiple daily injections.

Prevalence rates of arterial hypertension, hypercholesterolemia, and current smoking were 40.0%, 33.1%, and 28.6%, respectively. Concerning diabetes-related complications, 30.8% had diabetic nephropathy, of which 11% were at stage 2 nephropathy (ACR ≥ 300 mg/g and or an eGFR < 60 ml·min^−1^ per 1.73 m^2^). The prevalence of retinopathy at any stage was around 67.4%, of whom 38% were at stage 3 retinopathy. Peripheral neuropathy was found in 44%. 75% of the patients underwent screening for silent ischemic cardiac disease at the time of the first visit with only 3 positive screenings. The prevalence of neurovegetative disorders including gastrointestinal symptoms, bladder dysfunction, cardiovascular symptoms, and erectile dysfunction was found in 26.9%, 28.6%, 36.0%, and 29.1%, respectively.

According to Spallone et al. [1], fifty-five patients (31.4%) out of the 175 included did not have CAN at laboratory assessment. Sixty patients (34.2%) had possible CAN, 43 (24.6%) had a confirmed CAN, and 17 (9.7%) had severe CAN. An abnormal HR response to deep breathing was found in 61.2%, abnormal HR response to standing in 29.7%, abnormal Valsalva ratio or abnormal response to sustained hand grip in 12%, and, finally, orthostatic hypotension in 10.3%.

### 3.2. Risk Factors for CAN Progression over a Three-Year Period

One hundred and three (103) patients out of 158, with at least possible or more severe CAN at first laboratory assessment, had a second complete and analyzable testing. In sixty-two patients (51.7%), CAN severity remained stable or improved during the study period whereas worsening (change from possible to confirmed or from confirmed to severe CAN) was found in 41 patients (34.2%). The tests which became more frequently abnormal were the HR response to standing in 21% and the blood pressure response to sustained hand grip and Valsalva ratio in 14% and 15%, respectively. The blood pressure response to standing became abnormal in only 8% of the cases.

The characteristics of the two groups are shown in Table 2. A bivariate analysis indicates that age; gender; diabetes duration; cardiovascular-associated risk factors; morbidities including peripheral neuropathy, retinopathy, and nephropathy; and treatment modalities were not significantly different between the 2 groups. However, the mean BMI value was significantly lower (*p* = 0.047), and the exposure to selective serotonin reuptake inhibitors (SSRIs) was significantly higher (0.04) in patients from group II. In a multivariate analysis, both BMI and SSRI exposure were found to be independently related to CAN deterioration (Table 2).

### 3.3. Risk Factors for CAN Initiation over a Three-Year Period

Fifty-five (55) patients had no CAN at inclusion. Among them, 43 (78.2%) remained free from CAN at the end of the study, and 12 (21.8%) became positive on the second autonomic testing.

The clinical and biochemical characteristics of patients with persistent negative CAN and those who were positive for CAN upon the second reassessment were not statistically different (Table 3). A trend for higher exposure to diuretics in the group developing CAN (*n* = 2 patients) versus the group remaining free from CAN (*n* = 1 patient) was noticed but did not reach the level of significance (*p* = 0.053), and the very limited number of patients makes this finding nonrelevant.

## 4. Discussion

Previous studies provided information about cardiovascular autonomic neuropathy (CAN) prevalence in subjects with type 1 diabetes, but few of them raised the issue of the rate and predictors of CAN progression. The main results of the present study are that no clear risk factor was related to CAN onset and that factors associated with CAN progression from the mild stage are low BMI and SSRI exposure.

The interpretation of our results has to be made keeping in mind that the population included in this study is not representative of the global type 1 diabetic population. In fact, according to the strategy in our institution, and as recommended by French guidelines at the time of the study [9], only patients with at least one abnormal response to 3 bedside-performed screening tests were addressed for complete autonomic laboratory testing. Moreover, patients receiving beta-blockers, which could modify results of autonomic tests, were discarded, thus explaining the low rate of silent myocardial ischemia. Despite these potential biases, the observed frequency of CAN severity in this survey is coherent with previously published results [8, 10]. The selection strategy also explains that only a relatively reduced number of patients were free of cardiovascular autonomic neuropathy at the first assessment, thus making the evaluation of CAN incidence not realistic from our results. However, we observed that a reduced proportion of initially normal patients (21.8%) developed autonomic neuropathy within a 3-year period.

Our criterion for CAN progression was an increase of at least one point in the Spallone scoring. Indeed, very few studies investigated the progression rate of autonomic dysfunction in diabetes mellitus, and most of them concentrated on the prevalence of CAN. In these studies, the usual minimal score to diagnose CAN is 2 abnormal responses to cardiovagal tests to discard the false positive assessment of CAN [1]. In fact, the response to the tests composing the autonomic battery we used is highly dependent on the quality of patient performance. The choice of a one-point increase in autonomic score to define CAN progression was made in order to detect minimal changes accounting for progression. However, special attention was paid to the conditions in which autonomic testing was performed. In fact, all testings were performed at entry and at study end in the same laboratory by the same persons, and exactly the same devices were used in order to reduce the variability of the measured parameters. However, we cannot exclude that, despite these cautions, our experimental design could have led to some overestimation of the progression of CAN.

Other confusion biases potentially accounting for changes in the autonomic score at the second testing could result from changes in diabetes treatment. However, our data showed that cardiac autonomic neuropathy in long-standing type 1 diabetic patients is associated with the progression of autonomic dysfunction. During the DCCT/EDIC study, diabetes duration and degree of glycemic control were the dominant determinants of the risk progression of microvascular complications in type 1 diabetic patients [3]. However, CAN prevalence increased in both intensive and conventionally treated groups even if it was significantly lower in the former intensive group than in the former conventional group (28.9% versus 35.2%; *p* = 0.018) [3], thus suggesting that hyperglycemia is not the sole mechanism involved in CAN progression.

Regarding the progression of CAN, the present study demonstrates that 39.8% of the patients with mild autonomic dysfunction progress to a more advanced stage over a three-year period and that progression is related to low BMI and SSRI exposure but not to HbA1c level, disease duration, retinopathy, peripheral neuropathy, or nephropathy. Despite previous studies postulating that the progression of CAN is slow [11, 12] and linked to diabetes duration, blood glucose control, and microangiopathic complications [13–15], the nature of CAN progression predictors remains controversial. The progression of CAN has been shown to be positively correlated with low BMI, but, in the contrary, several studies reported obesity as a positive predictive factor for CAN progression in nondiabetic and type 1 and 2 diabetic patients [2]. Since most evidences indicate that low BMI is a marker of uncontrolled diabetes and thus a risk factor for CAN deterioration, our results have to be taken with caution and will need confirmation in specific future studies. In our study, the HbA1c level was not statistically different between patients that deteriorate their CAN and those who did not. This might be explained by the fact that the HbA1c level was measured at a point time related to the first CAN screening and does only show the blood glucose control over the last three-month period. Another possibility is that HbA1c is not a good indicator of blood glucose variability promoting hypoglycemic stress that was associated in a recent study with reduced heart rate variability independent of glycemic control as assessed by HbA1c [16]. Thus, it could be suggested that glucose variability rather than glucose levels contributes to cardiovascular autonomic dysfunction among adults with type 1 diabetes.

Our findings are surprising since diabetic CAN has been repeatedly reported to associate with nephropathy and since autonomic neuropathy is encountered in approximatively 60% of the patients with chronic kidney disease. The relationship between CAN and nephropathy in diabetes is rather complex and unclear. In fact, on one hand, CAN may be an aggravating factor of nephropathy [14]. A faster rate over 1 year of the progression of renal dysfunction was suggested in type 1 diabetic patients with autonomic dysfunction [17]. Nephropathy was found to be an independent risk factor for CAN prevalence [18]. On the other hand, autonomic neuropathy was not considered to be a progression promoter in diabetic nephropathy in a prospective observational follow-up study including 388 type 1 diabetic patients with (*n* = 197) and without (*n* = 191) diabetic nephropathy followed for 10.1 years [19]; in this study, there was no relationship between CAN and a faster decline in GFR. Finally, several authors have hypothesized that CAN is indirectly involved in the pathogenesis of diabetic nephropathy, via hemodynamic changes such as lack of nocturnal BP dipping (causing increased intraglomerular pressure resulting in albuminuria) and diurnal postural falls in BP (resulting in lower intraglomerular pressure) and endothelial dysfunction [19]. Others suggested that CAN is a strong predictor of nephropathy and participates in the progression of chronic kidney disease through deficient erythropoietin production and anemia [20–22].

We found that serotonin reuptake inhibitor exposure was higher in patients with CAN progression. This finding first suggests an indirect link between SSRIs and CAN worsening. In fact, even if SSRIs are not first-line recommended drugs for diabetic neuropathic pain, antidepressants are often used in diabetes, and, globally, an association between their use and CAN worsening could be related to a higher frequency of depressive symptoms in worsening patients. In fact, our data indicate that there is a significant difference (*p* = 0.04; OR: 4.18 [1.03–16.97]) in the frequency of SSRI use in patients with neuropathy in both patients remaining stable (6%) and worsening ones (20%). Secondly, it could suggest that SSRI drugs worsen diabetes equilibrium, thus favoring CAN progression. However, on the one hand, no differences in diabetes characteristics, including HbA1c, were found between stable and worsening patients, and, on the other hand, data from literature about effects of SSRIs on diabetes remain controversial. In young patients with type 1 diabetes, SSRIs have been found to be associated with negative clinical outcomes, an effect ascribed to the impact of comorbid depression on the quality of diabetes treatment [23]. By contrast, other studies have reported lower insulin requirement for the control of type 1 diabetes in patients receiving SSRIs [24]. Finally, another hypothesis could be the negative impact of SSRIs on autonomic nervous system activity. Data from the literature converge on neutral effects of SSRIs on autonomic function when compared to older tricyclic antidepressants [25]. SSRIs have also been proposed for the management of vasovagal syncope [26] or orthostatic hypotension in neurodegenerative disorders such as Parkinson's disease [27], but there are no data in orthostatic hypotension in diabetes mellitus.

## 5. Conclusion

Few studies raised the question about the initiation and progression of CAN in type 1 diabetic patients, which makes this paper original. The main findings are that no clear factors seem to be associated with CAN onset while its worsening is correlated with low BMI and SSRI exposure but not with classical clinical or biological disease parameters. Despite some limitations, resulting from the study design and the number of patients in subgroups which limit conclusions about any causal relation between CAN progression and other microvascular and macrovascular diabetes-related complications, these data suggest that mechanisms underlying the onset and worsening of autonomic dysfunction in type 1 diabetes are different. However, larger prospective studies with longer follow-up are needed to confirm these results.

## Figures and Tables

**Table 1:** Baseline characteristics of the cohort of type 1 diabetic patients.

	*N* = 175
*Demographic characteristics*	
Age (years)	50 ± 11
Women/men	76/99
Diabetes duration (years)	26 ± 11
HbA1c (%)	7.9 ± 1.2
BMI (kg/m^2^)	24.4 ± 3
*Complications and comorbidities*	
Nephropathy	54 (31%)
Retinopathy	118 (67%)
Peripheral neuropathy	77 (44%)
Hypertension	70 (40%)
Dyslipidemia	58 (33%)
Tobacco smoking	50 (29%)
Silent myocardial ischemia	3 (2%)
*Neurovegetative symptoms*	
Cardiovascular	63 (36%)
Digestive	47 (27%)
Erectile dysfunction	51 (52%)
Bladder dysfunction	50 (29%)

**Table 2:** Baseline clinical and biochemical characteristics of type 1 diabetic patients with mild CAN who remained stable (group I) or had CAN worsening (group II) for over a mean period of 3 years.

	Group I (*n* = 62)	Group II (*n* = 41)	*p* value	Logistic regression OR (95% CI)
Age (years)	50.69 ± 1.36	47.63 ± 1.57	0.15	
Males	39 (63%)	22 (54%)	0.35	
Diabetes duration (years)	26.32 ± 1.45	26.12 ± 1.62	0.92	
≤20	21 (34%)	13 (32%)	0.8	
21–30	22 (35%)	13 (32%)		
>30	19 (31%)	15 (37%)		
BMI (kg/m^2^)	25.07 ± 0.38	23.58 ± 0.5	0.018	
≤22.94	16 (26%)	20 (49%)	0.003	1
22.94–25.68	17 (27%)	15 (37%)		0.73 (0.28–1.95)
>25.68	29 (47%)	6 (15%)		0.15 (0.05–0.48)
HbA1c (%)	7.76 ± 0.13	8.08 ± 0.2	0.16	
≤7.4	23 (37%)	11 (27%)	0.25	
7.5–8.2	24 (39%)	14 (34%)		
>8.2	15 (24%)	16 (39%)		
DPN	27 (44%)	18 (44%)	0.97	
Nephropathy			0.72	
0	44 (71%)	26 (63%)		
1	11 (18%)	9 (22%)		
2	7 (11%)	6 (15%)		
Retinopathy			0.18	
0	22 (35%)	7 (17%)		
1	8 (13%)	8 (20%)		
2	10 (16%)	6 (15%)		
3	22 (35%)	20 (49%)		
Microalbuminuria (mg/24 h)	41 ± 12.32	163.16 ± 69.92	0.09	
≤7.0	23 (37%)	11 (27%)	0.44	
7.1–14.6	20 (32%)	13 (32%)		
>14.7	19 (31%)	17 (41%)		
Clearance (ml/min)	80.46 ± 2.02	79.26 ± 3.71	0.75	
≤73.87	20 (32%)	18 (44%)	0.37	
73.87–88.49	26 (42%)	12 (29%)		
>88.49	16 (26%)	11 (27%)		
Hypertension	25 (40%)	13 (32%)	0.37	
Tobacco smoking	18 (29%)	12 (30%)	0.91	
Dyslipidemia	17 (27%)	16 (39%)	0.21	
CSII			0.39	
0	28 (45%)	23 (56%)		
1	27 (44%)	16 (39%)		
2	7 (11%)	2 (5%)		
HMG-CoA inhibitors	16 (26%)	14 (34%)	0.36	
CEI/AT1 antagonists	26 (42%)	16 (39%)	0.76	
Antiaggregants	7 (11%)	5 (12%)	0.89	
Diuretics	5 (8%)	6 (15%)	0.27	
Calcium inhibitors	4 (6%)	3 (7%)	0.86	
SSRIs	4 (6%)	8 (20%)	0.04	4.18 (1.03–16.97)

Shown are means ± standard error of the mean. DPN: distal peripheral neuropathy; CV: cardiovascular; CSII: continuous subcutaneous and intraperitoneal insulin infusion; NC: not calculated. Data were analyzed according to Statistical Analysis.

**Table 3:** Baseline clinical and biochemical characteristics of type 1 diabetic patients without CAN at inclusion who remained stable (no CAN) or developed autonomic dysfunction (CAN) for over a mean period of 3 years.

	No CAN (*n* = 43)	CAN (*n* = 12)	*p* value	No CAN (*n* = 43) and not No CAN (*n* = 43)
Age (years)	52.12 ± 1.56	56.33 ± 3.56	0.23	
Males	27 (63%)	6 (50%)	0.42	
Diabetes duration (years)	25.47 ± 1.83	22.17 ± 2.66	0.38	
≤20	19 (44%)	6 (50%)	0.09	
21–30	8 (19%)	5 (42%)		
>30	16 (37%)	1 (8%)		
BMI (kg/m^2^)	23.74 ± 0.67	24.27 ± 0.71	0.69	
≤22.94	12 (28%)	4 (33%)	0.87	
22.94–25.68	17 (40%)	5 (42%)		
>25.68	14 (33%)	3 (25%)		
HbA1c (%)	8.07 ± 0.18	7.57 ± 0.37	0.21	
≤7.4	14 (33%)	6 (50%)	0.53	
7.5–8.2	14 (33%)	3 (25%)		
>8.2	15 (35%)	3 (25%)		
DPN	16 (37%)	5 (42%)	0.79	
Nephropathy			0.13	
0	32 (74%)	11 (92%)		
1	10 (23%)	0 (0%)		
2	1 (2%)	1 (8%)		
Retinopathy			0.89	
0	19 (44%)	6 (50%)		
1	6 (14%)	2 (17%)		
2	6 (14%)	2 (17%)		
3	12 (28%)	2 (17%)		
Microalbuminuria (mg/24 h)	44.71 ± 27.74	7.76 ± 1.32	0.48	
≤7.0	17 (40%)	8 (67%)	0.25	
7.1–14.6	12 (28%)	2 (17%)		
>14.7	14 (33%)	2 (17%)		
Clearance (ml/min)	85.31 ± 2.32	81.1 ± 4.68	0.4	
≤73.87	9 (21%)	5 (42%)	0.34	
73.87–88.49	15 (35%)	3 (25%)		
>88.49	19 (44%)	4 (33%)		
Hypertension	17 (40%)	6 (50%)	0.48	
Tobacco smoking	10 (23%)	4 (33%)	0.5	
Dyslipidemia	16 (37%)	3 (25%)	0.43	
CSII			0.6	
0	16 (37%)	6 (50%)		
1	19 (44%)	5 (42%)		
2	8 (19%)	1 (8%)		
HMG-CoA inhibitors	9 (21%)	1 (8%)	0.31	
CEI/AT1 antagonists	19 (44%)	7 (58%)	0.38	
Antiaggregants	7 (16%)	0 (0%)	0.13	
Diuretics	1 (2%)	2 (17%)	0.053	
Calcium inhibitors	4 (9%)	0 (0%)	0.27	
SSRIs	4 (9%)	0 (0%)	0.27	

DPN: distal peripheral neuropathy; CV: cardiovascular; CSII: continuous subcutaneous and intraperitoneal insulin infusion; NC: not calculated. Data were analyzed according to Statistical Analysis.
